# Inhibition of Nischarin Expression Promotes Neurite Outgrowth through Regulation of PAK Activity

**DOI:** 10.1371/journal.pone.0144948

**Published:** 2015-12-15

**Authors:** Yuemin Ding, Yuying Li, Lingchao Lu, Ruyi Zhang, Linghui Zeng, Linlin Wang, Xiong Zhang

**Affiliations:** 1 Department of Clinical Medicine, School of Medicine, Zhejiang University City College, Hangzhou, 310015, China; 2 Department of Physiology, School of Medicine, Quzhou College of Technology, Quzhou, 324000, China; 3 Department of Pathology, Jiaxing Second Hospital, Jiaxing, 314000, China; 4 Department of Basic Medical Sciences, School of Medicine, Zhejiang University, Hangzhou, 310058, China; Institute of Molecular and Cell Biology (IMCB), SINGAPORE

## Abstract

Nischarin is a cytoplasmic protein expressed in various organs that plays an inhibitory role in cell migration and invasion and the carcinogenesis of breast cancer cells. We previously reported that Nischarin is highly expressed in neuronal cell lines and is differentially expressed in the brain tissue of adult rats. However, the physiological function of Nischarin in neural cells remains unknown. Here, we show that Nischarin is expressed in rat primary cortical neurons but not in astrocytes. Nischarin is localized around the nucleus and dendrites. Using shRNA to knockdown the expression of endogenous Nischarin significantly increases the percentage of neurite-bearing cells, remarkably increases neurite length, and accelerates neurite extension in neuronal cells. Silencing Nischarin expression also promotes dendrite elongation in rat cortical neurons where Nischarin interacts with p21-activated kinase 1/2 (PAK1/2) and negatively regulates phosphorylation of both PAK1 and PAK2. The stimulation of neurite growth observed in cells with decreased levels of Nischarin is partially abolished by IPA3-mediated inhibition of PAK1 activity. Our findings indicate that endogenous Nischarin inhibits neurite outgrowth by blocking PAK1 activation in neurons.

## Introduction

The outgrowth of neurites from neurons during development or following an injury is controlled by both extra- and intra-cellular molecules [1]. These molecules eventually converge onto the growth cone cytoskeleton, where there is coordinated cross-talk between actin filaments and microtubules [2]. Many cytoskeleton-associated proteins regulate cytoskeletal remodeling within axons [3]. Their function is controlled by the activation or inhibition of particular signaling pathways, such as the Rho-GTPases (guanosine triphosphatases) pathway.

The Rho family of small GTPases, such as RhoA (Ras homologous member A), Rac1 (Ras-related C3 botulinum toxin substrate 1), and Cdc42 (cell division cycle 42), act as molecular switches in multiple signaling pathways, including those involved in regulating neuronal cytoskeletal dynamics [4,5], and are thus regarded as major regulators of axonal and dendritic growth [6,7]. These three family members all play a specific role in axonal and dendritic morphology. In general, GTP-bound RhoA is involved in growth cone collapse and axonal retraction in response to collapsing guidance cues [8,9]. Active GTP-bound Rac1 and Cdc42 enhance neurite outgrowth via phosphorylation and activation of p21-activated kinases (PAKs) [10,11], resulting in the formation of veil-like lamellipodia and finger-like filopodia in growth cones [12,13].

Nischarin was cloned from an embryonic mouse cDNA library by Alahari in 2000 [14]. In recent years, research has been focused on Nischarin’s ability to inhibit the migration and invasion of cancer cells. Alahari reported that Nischarin inhibits cell migration by selectively binding to the proximal transmembrane region of the integrin α5β1 subunit cytoplasmic tail [14,15]. Rac-induced cell migration is inhibited by Nischarin’s interactions with PAK1, which blocks PAK activation [14,16]. Nischarin also blocks PAK-independent Rac signaling [16,17]. Furthermore, Nischarin may regulate actin dynamics and inhibit cell invasion by inhibiting the activity of LIM Kinase 1 (LIMK1), a downstream effector of PAK1 [18]. However, these findings were obtained from non-neuronal cells. To our knowledge, no studies have investigated whether Nischarin affects the activation of Rho GTPases and regulates neurite outgrowth in neuronal cells. This is important because our recent findings suggest that Nischarin is highly expressed in neurons. We have previously observed co-localization of Nischarin and the cytoskeletal protein F-actin in Neuro-2a cell protrusions [19]. This suggests that Nischarin has a neuron-specific function. We hypothesize that Nischarin has a dynamic influence on cytoskeleton proteins, which are involved in cell motility processes, such as neurite outgrowth.

## Materials and Methods

### Ethical statement

The experimental procedures were approved by the Laboratory Animal Welfare and Ethics Committee of Zhejiang University. The animal experiment protocol approval no. is ZJU2014-429-01. Animals were handled in strict accordance with institutional guidelines. All efforts were made to minimize the number of animals used and their suffering.

### Lentiviral vectors

Four shRNAs of Nischarin (Nis-shRNA1-4), which target four common sequences of mouse and rat Nischarin (5’-CACAACTGTCGCAACCGC-3’, 5’-TGATGCCAAGACTGACCTT-3’, 5’-CCTCAGAGACAACCGGATT-3’ and 5’-AGCATTGCCGAGGTTGAAA-3’), and the corresponding scrambled shRNAs were synthesized by GeneCopoeia (Guangzhou, China) and subcloned into the lentiviral vector psiLv-U6 (GeneCopoeia, Guangzhou, China), which contains an eGFP coding sequence. To generate lentiviral particles, psiLv-U6 shRNAs were transfected into 293T Lentiviral packaging cellswith a Lenti-Pac™ FIV packing mix and an EndoFectin Lenti transfection reagent. Viral supernatants were harvested, concentrated, aliquoted, and stored at −80°C until use. Titers of the lentiviral stocks were assessed using 10-fold serial dilutions to transfect HEK293T cells together with an eGFP reporter to identify the infected cells.

### Neuro-2a cell culture and transfection

Neuro-2a cells were obtained from the Shanghai Cell Resource Center. Cells were cultured in Dulbecco's modified Eagle's medium (DMEM, Keyi, Hangzhou, China) supplemented with 10% fetal bovine serum (FBS, HyClone, Logan, UT, USA), and 1% v/v penicillin/streptomycin (Sigma, St. Louis, MO, USA). Cells were maintained in a humidified incubator with 5% CO_2_ at 37°C. For differentiation, the culture medium was switched to DMEM with 20 μM retinoic acid (RA, Sigma, St. Louis, MO, USA). To determine the effectiveness of the Nischarin shRNA, we used Lipofectamine 2000 (Invitrogen, Carlsbad, CA, USA) to transfect Neuro-2a cells with the plasmid for Nis-shRNA1-4 or control-shRNA. Forty-eight hours after transfection, knockdown of Nischarin mRNA and protein in cells was determined by using real time quantitative reverse transcription-polymerase chain reaction (RT-PCR) and western blot analysis, respectively.

### PC-12 cell culture and lentiviral infection

PC-12 cells (Shanghai Cell Resource Center) were cultured in RPMI 1640 medium (Keyi, Hangzhou, China) supplemented with 10% horse serum (HyClone, Logan, UT, USA), 5% FBS, 1% v/v penicillin/streptomycin and 2 mM L-glutamine (Invitrogen, Carlsbad, CA, USA). To knockdown Nischarin expression, PC-12 cells in six-well plates were grown to 80% confluency and infected with 10^5^ TU/well lentivirus. Four days after lentiviral infection, cells were differentiated with 50 ng/ml nerve growth factor (NGF, Sigma, St. Louis, MO, USA) for 24 h and the cells were subsequently examined for neuronal morphologies.

### Primary neuron culture and lentiviral infection

Sprague-Dawley (SD) rats were purchased from the Experimental Animal Centre of Zhejiang University (Hangzhou, China). Primary cultures of cortical or hippocampal neurons were prepared as previously described, with modifications [20]. In brief, the cortices or hippocampus of E18 rat embryos were dissected and dissociated with 0.25% trypsin (Invitrogen, Carlsbad, CA, USA) in oxygenated Hank's balanced salt solution (HBSS). Neurons (1×10^6^) were seeded onto dishes or coverslips coated with poly-D-lysine (10 mg/ml) in Neurobasal medium supplemented with 2% B27 (Invitrogen, Carlsbad, CA, USA) and 0.2 mM L-glutamine. Cytosine-D-arabinoside (10 μM Ara-C, Sigma, St. Louis, MO, USA) was added to cultures to block the proliferation of non-neuronal cells. One-half of the culture medium was changed every four days. The cultures were maintained in a 5% CO_2_ incubator at 37°C for 12–15 days. To examine the dendrite morphology, 0.5 ×10^5^ cortical neurons were plated onto poly-D-lysine coated coverslips. Lentiviral shRNAs were added to infect the neurons at 7 DIV (day *in vitro*). Three days after lentiviral infection, neurons were examined for dendrite morphology.

For cultures of mixed cortical neurons and astrocytes, cells were grown in Neurobasal medium supplemented with 2% B27 and 0.2 mM L-glutamine. After 10 days *in vitro*, glial cell division was stopped by exposure to 10 μM Ara-C for three days.

### Primary astrocyte culture

Astrocytes in primary culture were prepared from day 0 rat pups (0–12 h postnatal) as previously described [21]. Briefly, the cortex was dissected and trypsinized with 0.25% trypsin for 16 min, followed by trituration with 1% DNAse. Cells were seeded in poly-D-lysine-coated flasks and maintained in astrocyte medium supplemented with 15% N-2 (Invitrogen, Carlsbad, CA, USA) and 1% FBS. Culture medium was changed every third day. After nine days, the flasks were shaken for 12 h at 37°C to separate microglia from the mass of astrocytes. The adherent cells were re-plated on dishes and cultured for another week before being used in experiments.

### Protein extraction and western blot analysis

Neuronal cells were removed from dishes using a scraper and cold radioimmunoprecipitation (RIPA) lysis buffer (50 mM Tris-HCl pH 7.5, 150 mM NaCl, 50 mM NaF, 1% NP-40, 0.1% sodium deoxycholate, 1 mM sodium pyrophosphate) supplemented with protease inhibitor cocktail (Sigma, St. Louis, MO, USA). Cell lysates were centrifuged at 16,000 g for 10 min at 4°C. Protein concentrations were determined using a BCA assay kit (Beyotime, Shanghai, China). Cell lysates were separated by sodium dodecyl sulfate-polyacrylamide gel electrophoresis (SDS-PAGE) and transferred to a polyvinylidene difluoride (PVDF) membrane. After 2 h of blocking in 5% nonfat milk, blots were incubated overnight at 4°C with the following primary antibodies: anti-Nischarin (1:1000, BD Biosciences, San Jose, USA), anti-PAK1 and anti-PAK2 (1:500, Abcam, Cambridge, MA, USA), anti- phospho-PAK1 (Thr423)/PAK2 (Thr402)(1:1000, Cell Signaling Technology, Beverly, MA, USA) and anti-GAPDH (1:500, Good Here, Hangzhou, China). The next day, blots were washed in TBS with 0.1% Tween 20 (TBST) and incubated in secondary antibodies (conjugated with horseradish peroxidase, 1:1000, Jackson ImmunoResearch Laboratories, West Grove, USA) for 2 h at room temperature. Protein bands were visualized by enhanced chemiluminescence reagents (Amersham, Arlington Heights, USA) and exposed to X-ray film. Quantification of band intensity was performed using NIH ImageJ software (National Institutes of Health, Bethesda, MD). Values were normalized to GAPDH expression.

### Real time quantitative RT-PCR

Neuro-2a cells were transfected with Nis-shRNA1-4 or control-shRNA. Total RNA was extracted using TRIzol (Invitrogen, Carlsbad, CA, USA) according to the manufacturer’s protocol. cDNA was synthesized using a PrimeScript First-Strand cDNA synthesis kit (Takara, Dalian, China). For quantification of Nischarin mRNA expression (sense: 5’-ACCTGCAGTCAGTCAACGTC-3’, antisense 5’-CAGGAAGCAGTGTGTCAGGT-3’), real-time RT-PCR was performed using a CFX 96 Real-Time PCR Detection System (Bio-Red, Hercules, CA, USA), with GAPDH (5’-TGATTCTACCCACGGCAAGTT-3’, antisense 5’-TGATGGGTTTCCCATTGATGA-3’) as a control. Each 25 μl PCR mixture contained 12.5 μl SYBR Premix (Takara, Dalian, China), 400 nM primers, and 10 ng template. All reactions were performed in triplicate. PCR was run for 40 cycles with 5 s/95°C denaturation, 30 s/58°C annealing, and 30 s/72°C elongation. To verify the accuracy of the amplicon, a melting curve analysis was performed after amplification. Expression of Nischarin was assessed using the 2^-ΔΔCT^ formula.

### Multiple immunofluorescence staining

Primary cultured neurons and astrocytes were fixed in 4% paraformaldehyde for 10 min and processed using basic procedures as previously described [19]. Briefly, cells were permeabilized with 0.3% Triton X-100, blocked with 10% goat serum, and incubated with mouse anti-Nischarin monoclonal antibody (1:100) and rabbit anti-Map-2 polyclonal antibody (1:100, Santa Cruz Biotechnology, Santa Cruz, USA), rabbit anti-GFAP polyclonal antibody (1:2000, Cell Signaling Technology, Danvers, MA, USA), rabbit anti-PAK1 monoclonal antibody or anti-PAK2 monoclonal antibody (1:100, Abcam) overnight at 4°C, followed by incubation with anti-mouse FITC (1:100) and anti-rabbit Cy3 (1:100) for 2 h at room temperature. After rinsing with PBS, coverslips were mounted onto slides via a fluorescent mounting medium containing 4’, 6-diamidino-2-phenylindole (DAPI) to counterstain cell nuclei. Cells were imaged using an Olympus FluoView FV1000 confocal laser scanning microscope. The co-localization of Nischarin with Map-2, GFAP, PAK1 or PAK2 was performed using NIH Image J software.

### Live-cell imaging, cell counting and neurite length measurement

Neuro-2a cells were transfected with Nis-shRNA-3 or Ctl-shRNA and exposed to RA for 24 h. Cells in IPA3 group were treated with IPA3 (Selleck Chemicals, Houston, TX, USA) dissolved in DMSO at a final concentration of 10 μM for 24 h. To determine the number and length of neurites, phase images of five fields per well, containing at least 100 cells, were taken using an Olympus IX70 inverted optical microscopes. Processes longer than one cell body were considered neurites [4]. The length of neurite for each individual cell in these five fields of view was determined by manual tracing from the soma to the tip and was measured using the software of the NIH ImageJ. The mean length of the longest neurite and total neurite length for each cell were determined by statistical analysis. The number of neurite-bearing cells was counted and given as percentage of the total number of cells. For each graph, data on neurite length were generated from at least three independent experiments. For detailed description see Schwieger et al. 2015 [22].

PC-12 cells were also infected with lentiviral particles. Four days after the infection, PC-12 cells were exposed to NGF for 24 h. On the fifth day after infection, neurite outgrowth was quantified by imaging neuronal cells using an Olympus FV1000 confocal microscope. The longest neurites analyzed are processes from eGFP-positive cells. Time-lapse images were taken at three days post-infection in 45 min intervals to monitor the velocity of neurite extension in PC-12 cells.

To analyze dendrite development of the primary cultured neurons, 10 DIV cortical neurons were fixed and imaged with the FluoView FV1000 confocal microscope. Sholl analysis and quantification of dendritic length were performed using ImageJ as described previously [23].

### Co-Immunoprecipitation

For Nischarin/PAK binding, cortical neurons were lysed in RIPA lysis buffer with protease and phosphatase inhibitors. Cell lysates (500 μl, concentration 3 μg/μl) were incubated with anti-Nischarin antibody, anti-PAK1 or anti-PAK2 antibody at 4°C for 6 h. For controls, cell lysates were incubated with the same amount of immunoglobulin G (IgG, Sigma). After incubation with Protein G-Sepharose beads for 2 hours, the immunoprecipitates were washed three times with ice-cold lysis buffers, and the binding proteins were eluted with sample buffer. The general procedures for western blot were the same as described above.

### Statistical analysis

Data are presented as the mean ± SEM. Unless stated otherwise, one-way analysis of variance (ANOVA) with Student’s Newman-Keuls test were used for statistical comparison when appropriate. Differences were statistically significant at *p*<0.05.

## Results

### Nischarin is expressed in the cytoplasm of cortical neurons

**Fig 1:**
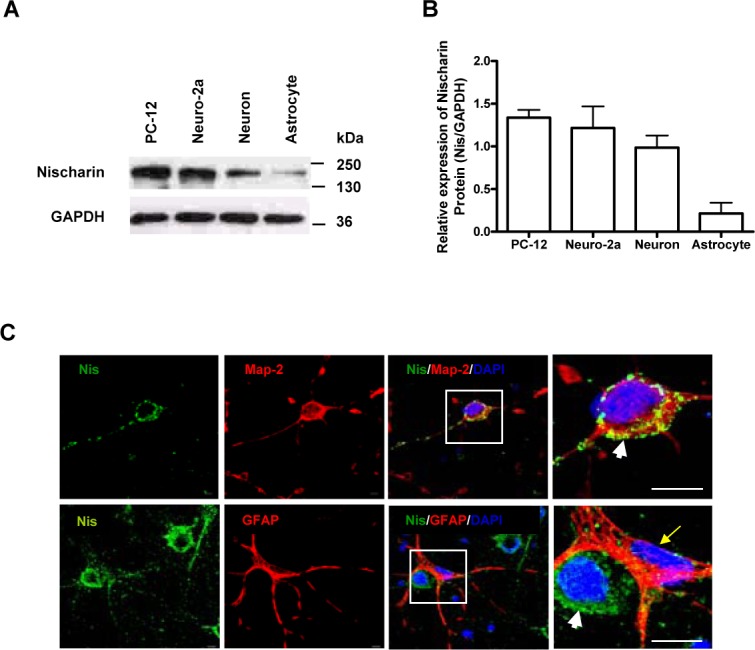
Cellular expression pattern of Nischarin in neurons. **(A, B)** Western blots revealed the expression of Nischarin protein in neuronal cell lines (PC-12 and Neuro-2a), neurons and astrocytes (n = 3). Expression levels of Nischarin were quantified by normalization against the housekeeping gene GAPDH. **(C)** Immunofluorescence staining was performed on pure cortical neuron cultures (top panel) and mixed cultures of neurons and astrocytes (bottom panel). Strong staining for Nischarin (green) co-localized with Map-2 (red) in the perinuclear region (arrowheads) and the dendrites of cortical neurons. No co-localization of Nischarin (green) and GFAP (red) was observed in the cytoplasm of astrocytes (arrow). Scale bars: 20 μm.

We have previously shown that Nischarin is expressed in different regions of the adult rat brain. However, the cellular expression pattern of Nischarin in the brain had not been elucidated. Here, we performed western blot analyses of total whole-cell lysates extracted from pure cultured cortical neurons, astrocytes, and two neuronal cell lines. Nischarin expression was enriched in both neurons and neuronal cell lysates but was relatively low in astrocyte lysates (Fig 1A and 1B). We determined the cellular distribution of Nischarin by double immunofluorescence staining a mix of cultured cortical neurons and astrocytes with antibodies raised against Nischarin (green), the neuronal marker Map-2 (red) or the astrocyte marker GFAP (red). Nischarin was expressed in the cytoplasm of neurons but not in astrocytes. High power images of cell bodies revealed that Nischarin expression overlaps with Map-2 in a punctate pattern near the perinuclear region and in the dendrites of cortical neurons. However, there was no similarity in the distribution pattern when using the reactive and radial glial marker, GFAP (Fig 1C). Nischarin was clearly visible in cortical neurons and absent from astrocytes.

### Nischarin inhibits neurite outgrowth in neuronal cells

**Fig 2:**
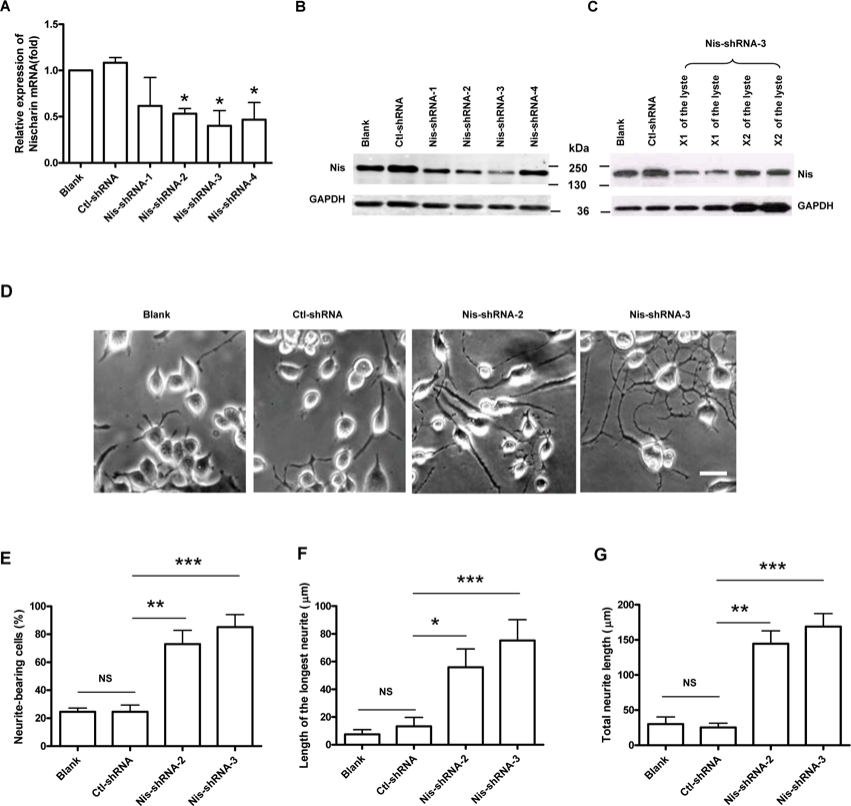
Knockdown of endogenous Nischarin expression promotes neurite outgrowth in Neuro-2a cells. Neuro-2a cells were transfected with Nis-shRNA1-4 or control-shRNA. Nischarin expression was determined by real time quantitative RT-PCR **(A)** and western blot assay **(B)**. Expression of the Nischarin gene and protein after transfection with Nis-shRNA1-4 decreased to some degree. The knockdown efficiency of Nis-shRNA-3 could reach up to ~60% inhibition. **(C)** The Nischarin signal came back to ~90% level when loading double amount of the lysates. Asterisks indicate a significant difference compared with control-shRNA (*p<0.05, n = 3). **(D)** Representative images of Neuro-2a cells 48 h after transfection with Nis-shRNA-2, Nis-shRNA-3 or control-shRNA. Cells were differentiated with 20 μM retinoic acid in DMEM for 24 h. For the analysis of the neurite length, pictures were taken with an inverted microscope from five fields of view per well. The neurite length for each individual cell was determined by manual tracing and measured using NIH ImageJ software. Neurites were defined as a process with lengths equivalent to one diameters of a cell body. The percentage of neurite-bearing cells was calculated from the total number of counted cells (n = 3, ~1200 cells measured). Scale bar: 20 μm. Suppressing expression of Nischarin increased the number of neurite-bearing cells **(E)**, the mean length of the longest neurite **(F)**, and the total neurite length **(G)**. Asterisks indicate significant differences from the control shRNA cells (*p<0.05). Data presented are the mean ± SEM.

Our previous studies revealed that Nischarin overlaps F-actin in the filopodia-like protrusions of Neuro-2a cells, raising the question of whether Nischarin plays a role in regulating neurite outgrowth [19]. To address the issue, we constructed four shRNAs (Nis-shRNA 1–4) targeting different sequences of rat and mouse Nischarin and their scrambled shRNA (ctl-shRNA). Neuro-2a cells were transfected with Nis-shRNA1-4 or ctl-shRNA and transfection efficiency was determined by eGFP expression 48 h after transfection (data not shown). Expression levels of Nischarin were determined by real time quantitative RT-PCR (Fig 2A) and western blot assay (Fig 2B). The results revealed that expression of the Nischarin gene and protein after transfection with Nis-shRNA1-4 decreased to some extent. Nis-shRNA-3 was the most effective construct for knockdown of endogenous Nischarin expression with a ~60% efficiency compared with other constructs. It was confirmed by doubling the amount of lysates loading (in the Nis-shRNA-3) and the Niscarin signal came back to ~90% level (Fig 2C).

Subsequently, Nis-shRNA-2 and Nis-shRNA-3 were used to suppress the endogenous expression of Nischarin in Neuro-2a cells after which, cell morphology was observed under a phase contrast microscope. Nis-shRNA-mediated Nischarin knockdown promoted remarkable neurite outgrowth (Fig 2D). Approximately 80% of the Nischarin-suppressed Neuro-2a cells extended neurites, whereas only 20–30% of normal or control-shRNA transfected cells bore neurites (Fig 2E). Furthermore, both the mean length of the longest neurite and the total neurite length were significantly increased in Nischarin knockdown cells compared with those expressing scrambled shRNAs (Fig 2F and 2G). These findings suggest that endogenous Nischarin inhibits neurite extension in Neuro-2a cells.

**Fig 3:**
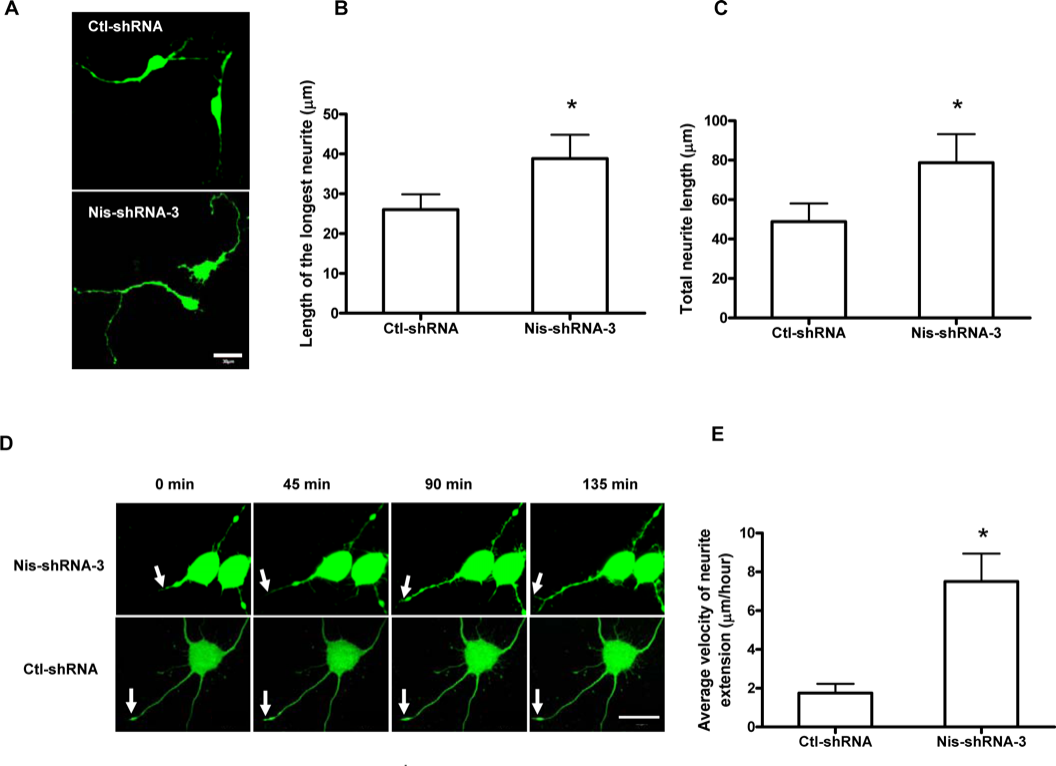
Knockdown of Nischarin expression promotes neurite outgrowth in PC-12 cells. **(A)** Fluorescence images of PC-12 cells five days after infection with Nis-shRNA-3 or control-shRNA lentivirus. Four days after the infection, PC-12 cells were exposed to NGF for 24 h. Scale bar: 20 μm. **(B, C)** Quantification of neurite outgrowth five days after lentivirus infection. The neurites analyzed are processes from eGFP-positive cells under a confocal microscope (n = 3, ~200 cells measured). Suppressing Nischarin expression increases the mean length of the longest neurite and the total length of neurites per cell. Asterisks indicate significant differences compared with control-shRNA cells (*p<0.05, **p<0.01). Data presented are the mean ± SEM. **(D)** Images from time-lapse recordings of PC-12 cells after infection by Nis-shRNA-3 or control-shRNA lentivirus. Time-lapse images were taken at three days post-infection in 45 min intervals to monitor the velocity of neurite extension in PC-12 cells. Scale bar: 20 μm. **(E)** Quantification of the average velocity of neurite extension.

To confirm the inhibitory effect of Nischarin on neurite outgrowth, we investigated the neurite morphology of PC-12 cells where Nischarin expression was suppressed. Because of their relatively low transfection efficiency, PC-12 cells were infected by lentiviral Nis-shRNA-3. We used live imaging to monitor neurite outgrowth in the eGFP positive cells (Fig 3A). Consistently, the mean length of the longest neurite and the total neurite length increased in Nischarin-suppressed cells compared with those expressing scrambled shRNA five days after lentivirus infection (Fig 3B and 3C). Time-lapse recordings also show an increased average velocity of neurite extension in Nischarin-suppressed cells (Fig 3D and 3E).

### Nischarin inhibits dendrite elongation in cortical neurons

**Fig 4:**
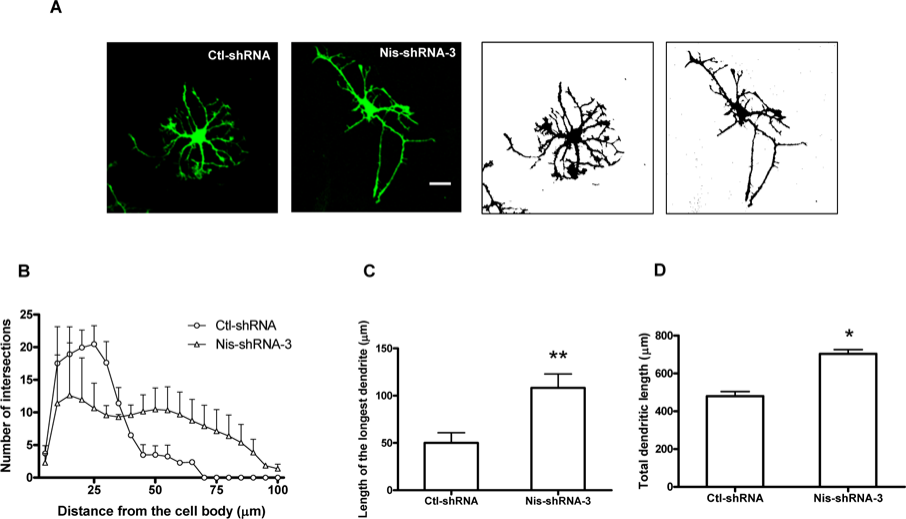
Knockdown of Nischarin expression promotes dendrite elongation in cortical neurons. **(A)** Cortical neurons were infected with lentiviral control-shRNA or Nis-shRNA-3 at 7 DIV and dendrite morphology was examined at 10 DIV. Scale bar: 20 μm. **(B)** Number of dendritic intersections within 100 μm of the cell body determined by Sholl analysis. Suppressing Nischarin expression increases the mean length of the longest dendrite **(C)** and total length of dendrites per cell **(D)**. Asterisks indicate a significant difference from control shRNA cells (*p<0.05, **p<0.01, n = 3, ~60 neurons measured). Data presented are the mean ± SEM.

To verify its effect on dendrite development in cortical neurons, we silenced Nischarin expression by lentiviral Nis-shRNA at 7 DIV, the stage where extensive dendrite arborization and elongation occurs. The morphology of infected cortical neurons was examined at 10 DIV (Fig 4A). In Nischarin-deficient neurons, there were fewer dendritic intersections at 20–35 μm from the cell body compared with control neurons. However, there were more intersections 40 μm or further from the cell body in Nischarin-deficient neurons compared with controls, as assessed by Sholl analysis (Fig 4B). Moreover, the mean length of the longest dendrite and the total length of the dendrites increased significantly in Nischarin-deficient neurons (Fig 4C and 4D). These results suggest that although Nischarin may not be critical for dendritic branching and arborization, it may play an important role in the initial elongation of dendrites.

### Nischarin-regulated neurite outgrowth occurs via interaction with and activation of PAK

Alahari and coworkers previously showed that Nischarin interacts with PAK1 in the CHO cells and the PC-12 cells to regulate cell migration [17]. They detected the exogenous protein interaction in Cos-7 cells by transfected with plasmids of Myc-tagged Nischarin (or its fragments) and V5-tagged PAK1. However, the endogenous protein interaction and the localization data in neurons are not valid

**Fig 5:**
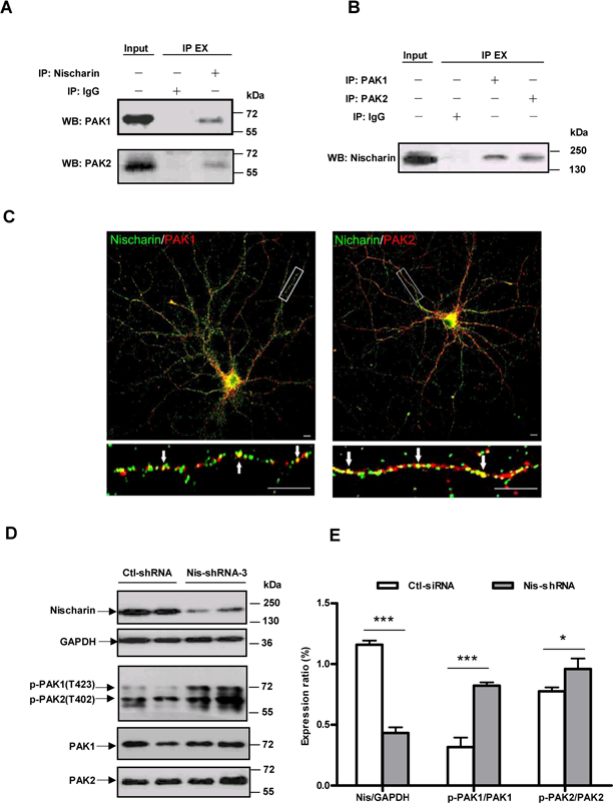
Nischarin interacts with PAK1/2 and regulates PAK activity. Lysates (500 μl, concentration 3 μg/μl) of rat cortical neurons were incubated with an anti-rabbit PAK1 polyclonal antibody, an anti-rabbit PAK2 polyclonal antibody or an anti-mouse Nischarin monoclonal antibody and their IgG then precipitated with G-Sepharose protein beads. **(A)** The Nischarin immunoprecipitates were blotted with anti-PAK1 antibody (top panel) or with anti-PAK2 antibody (bottom panel). **(B)** PAK1 or PAK2 immunoprecipitates were blotted with anti-Nischarin antibody. IgG was used as the negative control and 5% cell lysates (input) was added in the gel in each experiment to be a positive control. IP EX: immunoprecipitation extract. **(C)** Immunofluorescence staining was performed on primary cultured hippocampal neurons (10 DIV). Strong staining for Nischarin (green) co-localized both with PAK1 (red, left panel) and PAK2 (red, right panel) in the perinuclear region and the dendrites (arrows). Scale bars: 10 μm. **(D, E)** Nischarin-shRNA effectively suppresses the expression of Nischarin protein and stimulates the phosphorylation of both PAK1 and PAK2, in Neuro-2a cells (n = 3). Asterisks indicate significant differences between ctl-shRNA and Nis-shRNA (*p<0.05, ***p<0.001, n = 3). Data presented are the mean ± SEM.

In order to investigate if the protein interaction was endogenous in neurons, we performed endogenous co-IP assays. Nischarin was immunoprecipitated from the cortical neuron lysates using an anti-Nischarin antibody, the PAK1 or PAK2 in the precipitates were detected by western blot analysis. IgG was used as the negative control. The results showed that pull-down of Nischarin resulted in the co-immunoprecipitation of both PAK1 and PAK2 (Fig 5A). Reciprocal immunoprecipitation of PAK1 or PAK2 with the anti-PAK1 or anti-PAK2 antibody co-immunoprecipitated Nischarin (Fig 5B). The unrelated protein IgG bound neither Nischarin nor PAK in these assays. The findings indicated that interaction between Nischarin and PAK1 or PAK2 could happen at endogenous protein levels.

Consistently, double immunofluorescence study revealed extensive co-localization of Nischarin and PAK1, particularly in the cell body and the dendrites of the hippocampal neurons (Fig 5C, left panel), indicating the existence of interaction between Nischarin and PAK1. Similarly, immunofluorescence study also revealed a co-localization of the expression of Nischarin and PAK2 (Fig 5C, right panel).

To determine if the interaction between Nischarin and PAK regulates PAK activity, we examined the phosphorylation of PAK1 or PAK2 by reducing endogenous levels of Nischarin in cortical neurons. The Nischarin-shRNA caused a substantial reduction in the amount of endogenous Nischarin. This reduction dramatically stimulated the phosphorylation and activation of both PAK1 and PAK2 (Fig 5D and 5E).

**Fig 6:**
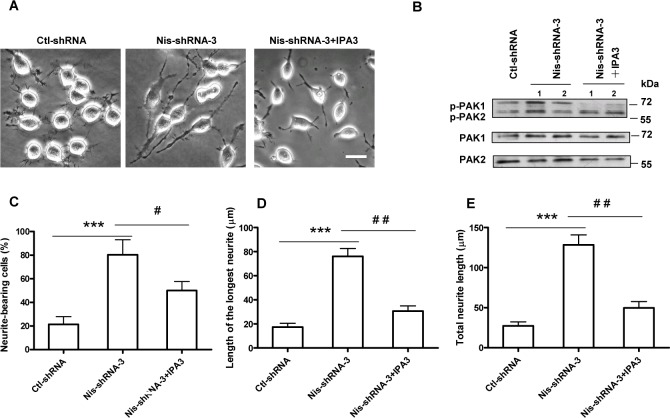
PAK1 activity is important for Nischarin-regulated neurite outgrowth in Neuro-2a cells. **(A)** Representative images of Neuro-2a cells transfected with Nis-shRNA-3 and treated with the PAK1 inhibitor IPA3. Scale bar: 20 μm. **(B)** Neuro-2a cells were transfected with Nis-shRNA-3 and treated with IPA3. Phosphorylation of PAK1/2 was determined by western blot assay. IPA3 treatment reversed the increases in the number of neurite-bearing cells **(C)**, the mean length of the longest neurite **(D)**, and total length of neurite per cell **(E)** in Nischarin-suppressed cells. Asterisks indicate significant differences between ctl-shRNA and Nis-shRNA (***p<0.001, n = 3), or between Nis-shRNA and Nis-shRNA+IPA3 (^#^ *p*<0.05, ^##^ *p*<0.01, n = 3). Data presented are the mean ± SEM.

To further investigate whether PAK activity is important for neurite outgrowth, we treated Nischarin-suppressed Neuro-2a cells with IPA3 (10 μM), a PAK1-specific inhibitor which substantially attenuated PAK1 phosphorylation, but not PAK2 (Fig 6A and 6B). Knockdown of Nischarin typically promotes an increase in the number of neurite-bearing cells (Fig 6C), the mean length of the longest neurite (Fig 6D), and the total length of the neurites (Fig 6E). Treatment with IPA3 blocked all of these effects within 24 h. The inhibition of neurite outgrowth when PAK1 activity was blocked by IPA3 confirms that PAK1 activity is required.

## Discussion

We previously investigated the distribution of Nischarin in the rat brain and found that it is highly expressed in the mature neurons (e.g. layers IV-V pyramidal cells of the cerebral cortex and the CA region of the hippocampus), whereas it is poorly expressed in the hippocampal DG region and the olfactory bulb, where the new-born cells exist [19]. Differential distribution of Nischarin in the brain reveals its regional characteristics and clear cellular selectivity. To further study the cellular and subcellular localization of Nischarin, we investigated the distribution of Nischarin in primary cultures of neurons and astrocytes. Nischarin is mainly distributed in the cytoplasm of neurons, especially in the perinuclear region and dendrites. In astrocytes, which make up a large volume of the brain, there is little to no expression of Nischarin in the cytoplasm. These findings are consistent with work by Wu et al., who recently reported the expression pattern of Nischarin protein in the inflamed cerebral cortex of rats. After LPS treatment, Nischarin expression is increased in the cytoplasm of cortical neurons but not in oligodendrocytes or astrocytes [24].

To explore the effect of Nischarin on neurite outgrowth, we measured the length and number of neurites after suppressing Nischarin expression by shRNA. Compared with the negative control group, suppressing the expression of endogenous Nischarin protein significantly increases the percentage of cells with neurites and the length of neurites in neural cell lines. In addition, we observed accelerated elongation of neurites. In cortical neurons, silencing Nischarin expression promotes dendrite elongation but not arborization. Dendritic development is a complex process, which generally can be divided into three stages: (i) initiation (elongation), (ii) branching (arborization), and (iii) stabilization [25]. We previously found that Nischarin is selectively expressed in the mature neurons, but not in the immature ones. We speculated that the absence of Nischarin in the early stage enables the unbranched dendrites to extend. As the expression level of Nischarin elevates in the mature neurons, dendritic elongation stops and arborization starts. These findings strongly suggest that Nischarin protein is able to precisely regulate dendrite growth during the different phases of development.

Studies using cancer cells have made great progress towards understanding the molecular mechanisms underlying how Nischarin functions [18,26,27,28,29]. In regulating cancer cell migration, the main targets for Nischarin are the Rho-GTPase signaling pathway and its downstream signaling molecules [17]. PAKs are the main effectors of Rac1 and Cdc42 GTPases, both of which play an important role in neurite outgrowth [30]. Rac1 and Cdc42 bind to and activate PAK [10,31] and regulate cytoskeleton dynamics in neurons [32]. In NGF treated PC-12 cells, endogenous PAK is redistributed to the membrane and induces neurite outgrowth, regardless of its kinase activity [33]. When N1E-115 neuroblastoma cells are in presence of repulsive molecules, membrane-targeted PAK is required for neurite outgrowth to overcome negative guidance cues and cross the repulsive barrier [34]. Alahari et al. [17] have previously reported that Nischarin co-localizes with PAK1 in membrane ruffles, structures known to be involved in cell motility. Besides, they further examined the interaction between Nischarin and other PAK isoforms and reported that Nischarin also interacts with PAK4 and PAK5. Here, we showed a direct association between endogenous Nischarin and PAK1/2 in cortical neurons. Since PAK plays a crucial role in the regulation of cell morphology as described above, our finding strongly suggests that Nischarin may regulate neurite outgrowth via its physical interaction with PAK.

In addition to its localization, the kinase activity of PAK may be important for regulating neuronal outgrowth, due to its essential role in regulating actin cytoskeleton dynamics [32,35]. Active PAK1 induces an increase in the number of dendrites in cortical neurons [36]. PAK1 also associates with Rac1 and the cyclin dependent kinase 5 (Cdk5) to form a complex in neuronal growth cones and induce neurite outgrowth [37,38]. Similarly, PAK2 has pivotal roles in a wide range of cellular activities including cell differentiation. Shin et al.[39,40] reported that PAK2 mediates neurite outgrowth via Rac1 GTPase and RhoGDI1 in PC12 cells. Interestingly, we found that Nischarin negatively regulates phosphorylation of both PAK1 and PAK2, raising the question whether PAK activity is the key factor for Nischarin to regulate neurite outgrowth. To address this issue, we used IPA3 to specifically inhibit PAK1 activity. This treatment abolished the neurite outgrowth normally induced by Nischarin suppression. Thus, our data indicate that Nischarin regulates neurite growth through PAK1 activity.

In conclusion, Nischarin protein is selectively expressed in neurons but not in astrocytes. In neurons, endogenous Nischarin binds to PAK1/2 and negatively regulates neurite outgrowth by blocking PAK1 activation.
